# Assessment of the optimal cutoff value of fasting plasma glucose to establish diagnosis of gestational diabetes mellitus in Chinese women

**DOI:** 10.1038/s41598-019-52509-7

**Published:** 2019-11-05

**Authors:** Bing Yan, Ya-xin Yu, Yin-ling Chen, Wei-juan Su, Yin-xiang Huang, Mu-lin Zhang, Bing-kun Huang, Li-li Han, Hai-qu Song, Xue-jun Li

**Affiliations:** 1 Xiamen Diabetes Institute, Xiamen, China; 2 Department of Endocrinology and Diabetes, The First Affiliated Hospital of Xiamen University, Xiamen, China; 3 Medical College of Xiamen University, Xiamen, China; 4 Xinglin Branch, The First Affiliated Hospital of Xiamen University, Xiamen, China; 5 Fujian Medical University, Fuzhou, China

**Keywords:** Diseases, Gestational diabetes

## Abstract

Our aim is to assess the optimal cutoff value of fasting plasma glucose (FPG) in Chinese women at 24–28 weeks’ gestation by performing oral glucose tolerance test (OGTT) to improve diagnostic rate of gestational diabetes mellitus (GDM). Data were derived from the Medical Birth Registry of Xiamen. A FPG cutoff value of 5.1 mmol/L confirmed the diagnosis of GDM in 4,794 (6.10%) pregnant women. However, a FPG cutoff value of 4.5 mmol/L should rule out the diagnosis of GDM in 35,932 (45.73%) pregnant women. If we use this cutoff value, the diagnosis of GDM to about 27.3% of pregnant women will be missed. Additionally, a 75-g OGTT was performed in pregnant women with FPG values between 4.5 and 5.1 mmol/L, avoiding the performance of formal 75-g OGTT in about 50.37% pregnant women. Meanwhile, according to maternal age and pre-pregnancy BMI categories, with FPG values between 4.5 mmol/L and 5.1 mmol/L, which had high sensitivity, to improve the diagnostic rate of GDM in all groups. Further researches are needed to present stronger evidences for the screening value of FPG in establishing the diagnosis of GDM in pregnant women.

## Introduction

Gestational diabetes mellitus (GDM) is defined as any degree of glucose intolerance with first recognition or onset during pregnancy^1^, which is an increasing public health problem. Worldwide, a total of 21.3 million women experienced hyperglycemia during pregnancy and nearly 86.4% of pregnant women were diagnosed to have GDM in 2017^2^. Furthermore, studies indicate that compared with European women, Asian women have a higher incidence of GDM^3,4^. Chinese pregnant women experience an elevated incidence of GDM due to China’s rapid economic and social development, which led to the change of lifestyle in the past several decades^5^. In 2015, a total of 2.90 million pregnant women suffered from GDM in China^6^, resulting in the implementation of one-child policy by the Chinese government.

Consequently, GDM has become a major public health problem because of China’s limited social and medical resources. Hence, in China, the health burden of GDM is significant. One study reported that the cost of pregnant women with GDM was ¥ 6,677.37 more than that of pregnant women without GDM. As a result, in 2015, the total burden cost of GDM was calculated to be ¥ 19.36 billion^6^, which is equal to 0.5% of the total public expenditure for healthcare and medical and family planning in China^7^. Due to the silent nature of GDM, an oral glucose tolerance test (OGTT)was performed to establish the diagnosis of GDM in pregnant women at 24–28 weeks’ gestation^8^.

Considering that the huge economic cost and social resource consumption for the diagnosis of GDM by implementing OGTT, not all pregnant women can perform a 75-g OGTT in some remote rural areas because of China’s limited medical and social resources^9,10^. Therefore, we can choose to establish a relatively reliable FPG threshold in OGTT as the reference value, so as to judge whether to continue to carry out the remaining experiments in OGTT, and ultimately achieve resource saving and improve diagnostic rate. One study reported that when the risk of GDM was between 1.0% and 4.2%, performing fasting plasma glucose (FPG) test followed by OGTT was the cost-effective method^11^. Moreover, another research compared the costs of the following three strategies in establishing the diagnosis of GDM: 100-g OGTT, 75-g OGTT, and FPG test of the OGTT. Consequently, the research revealed that performing FPG test was the ideal method^12^. Therefore, performing FPG test is recommended to establish the diagnosis of GDM at 24–28 weeks’ gestation. Additionally, initially performing an FPG test prevents the need of performing many subsequent OGTTs, hence reducing the health burden of the Chinese societies and families.

The advantages of performing FPG test to establish the diagnosis of GDM include the following: the test is cheap, reproducible, and reliable and has no vomiting response, response that is usually evident when performing OGTT or the glucose challenge test. FPG test can be performed in pregnant women who were unable to tolerate glucose-containing drinks. FPG test was performed to establish the diagnosis of GDM^13^. Zhu *et al*. reported that during the first prenatal visit, increased FPG level was strongly associated with GDM at 24–28 weeks’ gestation in China^14^. However, there were only two similar studies that assessed the sensitivity and specificity of FPG value in establishing the diagnosis of GDM at 24–28 weeks’ gestation in China, stressing that if the FPG cutoff value was between 4.4 mmol/L and 5.1 mmol/L, the pregnant women should undergo OGTT^14,15^. Whereas, one of the disadvantages in the above studies was that the data from the above study were derived from a hospital that consisted of a small sample size due to the following reason: rural residents rarely visited to the general hospital.

Chinese women tend to get pregnant at young ages^16^ is different from the other countries or healthcare systems, especially in developed countries, where women get pregnancy at older ages^17^. As well, pre-pregnancy body mass index (BMI) is a known risk factor for GDM^18,19^. In addition, studies revealed pre-pregnancy BMI might be as a predictor for GDM. Whereas, compared to population in other races, the BMI levels of Asians might be generally lower, which was contributed to ethnic differences^20,21^.

Therefore, this study aimed to assess the sensitivity and specificity of FPG value to establish the diagnosis of GDM based on maternal age and pre-pregnancy BMI categories in China with a registered data and to improve the diagnostic rate of GDM, hence avoiding the performance of a number of OGTTs and consequently reducing the health burden of the Chinese societies and families.

## Materials and Methods

### Study design

The Medical Birth Registry of Xiamen (MBRX) recorded the results of a 75-g OGTT for pregnant women and implemented the International Association of Diabetes and Pregnancy Study Group (IADPSG) criteria (one-step approach). Over 7 years, from March 1, 2011, to March30, 2018, pregnant women at 24–28 weeks’ gestation who were registered at the MBRX underwent the 75-g OGTT in Xiamen. According to the GDM diagnostic criteria, the venous plasma glucose values were recorded and analyzed. We created the receiver operating characteristic (ROC) curves to detect the FPG value and subsequently to establish the diagnosis of GDM based on the result of the 75-g OGTT, which is the standard method, and each point as a screening node was analyzed.

### Definition

The diagnosis of GDM could be established by the Ministry of Health in China according to IADPSG^22^ when any of the following FPG values were met or exceeded: 0 h, greater than or equal to5.1 mmol/L; 1 h, greater than or equal to 10.0 mmol/L; and 2 h, greater than or equal to 8.5 mmol/L. BMI was calculated on account of self-reported weight and measured height. According to World Health Organization recommendations for Asian population^23^, the pregnant women were classified into four groups: underweight, BMI <18.5 kg/m^2^; normal weight, 18.5–23.9 kg/m^2^; overweight, 24.0–27.9 kg/m^2^; and obesity, ≥28 kg/m^2^. Maternal age was classified into four groups: ≤25 years; 26–30 years; 31–35 years; and >35 years.

### Informed consent and ethics statements

The ethics committee of the First Affiliated Hospital of Xiamen University approved our study waived the need for informed consent, which composed and worked in accordance with the Chinese GCP and relevant regulations. The application number was KYH2018-007. In addition, this study was carried out in accordance with the rules of the Declaration of Helsinki of 1975, revised in 2013.

### Statistical analysis

The characteristics of study population were analyzed by SPSS version 17.0 statistical software (SPSS Inc., IL, USA). Continuous variables were showed as Median (min-mix). Discontinuous variables were expressed as n (%), which were analyzed by Chi-square (χ^2^) test. This study compared FPG cutoff values across pre-pregnancy BMI and maternal age categories. Receiver operating characteristic (ROC) curve analysis was conducted to identify the diagnostic power of FPG value of OGTT at pre-pregnancy in predicting development of GDM. The level of statistical significance is set at 0.05.

## Results

### Characteristics of study population

**Figure 1:**
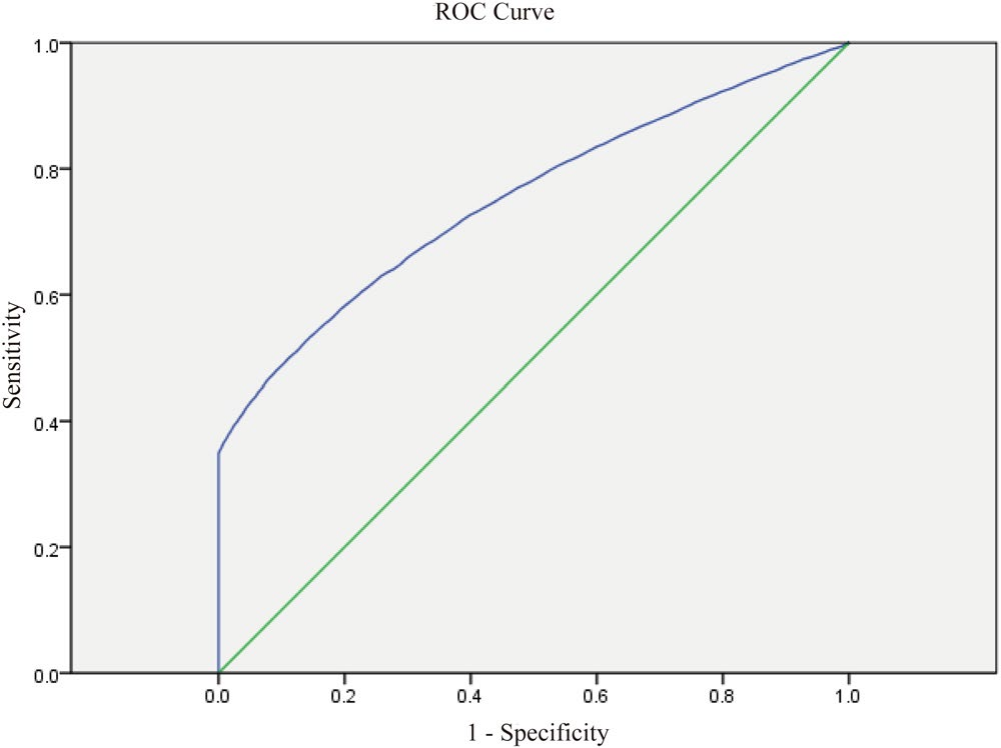
The receiver operating characteristic (ROC) curve of the participants in this study (the area under the ROC curve was 0.752; 95% CI, 0.747–0.757; SE,0.003; *P* < 0.001).

A total of 78,572 pregnant women (age range, 18–53 years old) registered at the MBRX underwent a 75-g OGTT. Most of the pregnant women (51,600) had education levels higher than 9 years. Additionally, the parity of the 36,869 (53.00%) pregnant women was more than two times (Table 1). A total of 13,658 pregnant women were diagnosed to have GDM on account of maternal age and pre-pregnancy BMI categories based on the IADPSG criteria. The results of performing a 75-g OGTT in order to establish the diagnosis of GDM revealed that the following pregnant women have met or exceeded the FPG values: 0 h (≥5.1 mmol/L), 2,753 pregnant women; 1 h (≥10.0 mmol/L), 3,088 pregnant women; and 2 h (≥8.5 mmol/L), 3,257 pregnant women. The diagnostic rates of GDM were 18.8%, 21.07%, and 22.2%, respectively. Furthermore, the diagnostic rate of GDM was 35.7% following the FPG criteria in the OGTT. To assess the FPG value of OGTT to establish the diagnosis of GDM at 24–28 weeks’ gestation, the ROC curves are shown in Figure 1. The area under the ROC curve was 0.752 (95% CI, 0.747–0.757; SE, 0.003; *P* < 0.001).

**Table 1:** Characteristics of study population.

	N	Median (min-max) or n (%)
Age, years	77,859	28 (18–53)
BMI, kg/m^2^	77,859	20.6 (13.3–44.4)
Systolic blood pressure, mmHg	51,788	107 (70–160)
Diastolic blood pressure, mmHg	51,794	65 (40–105)
Education, No.	68,066	100%
≤9 years	16,900	24.83%
>9 years	511,600	75.17%
Family history of diabetes, No.	73,670	100%
Yes	1,825	2.48%
No	71,845	97.52%
Family history of hypertension, No	73,670	100%
Yes	3,849	5.22%
No	69,821	94.78%
Fasting glucose in first trimester, mmol/L	46,183	4.7 (2.9–8.7)
OGTT at week 24–28, mmol/L		
Fasting glucose	78,572	4.5 (3.0–14.3)
1-h glucose	78,572	7.8 (20–23.2)
2-h glucose	78,572	6.6 (2.5–23.9)
Parity, No.	69,559	100%
1	32,690	47.00%
≥2	36,869	53.00%

BMI, body mass index; OGTT, oral glucose tolerance test.

### The overall association between FPG cutoff value and GDM diagnosis

FPG value was more than 5.1 mmol/L, the diagnosis of GDM (6.10%) in pregnant women was confirmed. As shown in Table 2, with the cutoff FPG value of 4.5 mmol/L, 72.7% of pregnant women were diagnosed to have GDM with specificity of 0.600. If the FPG value of 4.5 mmol/L was the cutoff value to identify who should undergo the 75-g OGTT, then 50.37% (44.27% with values were less than 4.5 mmol/L plus 6.10% with values were greater than 5.1 mmol/L) of pregnant women could avoid the performance of a 75-g OGTT with the probability that 27.3% of pregnant women with GDM may miss to undergo a 75-g OGTT. If the FPG value of 4.4 mmol/L or 4.3 mmol/L serves as the cutoff value, the missed percentage of pregnant women who should undergo a 75-g OGTT would be 20.2% or 14.2% with the specificity was 0.474 and 0.350 respectively.

**Table 2:** Fasting glucose plasma cutoff values of gestational diabetes mellitus diagnosis.

Cut point (mmol/L)	At or above the value, n (%)	Sensitivity	Specificity	FPR	FNR	Youden index	PLR	NLR	PPV	NPV
4.0	72457 (92.22)	0.968	0.088	0.032	0.912	0.056	1.061	0.364	0.926	0.189
4.1	67954 (86.49)	0.943	0.152	0.057	0.848	0.095	1.112	0.375	0.877	0.294
4.2	61580 (78.37)	0.906	0.242	0.094	0.758	0.148	1.195	0.388	0.813	0.415
4.3	53896 (68.59)	0.858	0.350	0.142	0.650	0.208	1.320	0.406	0.743	0.530
4.4	45030 (57.31)	0.798	0.474	0.202	0.526	0.272	1.517	0.426	0.671	0.636
4.5	35932 (45.73)	0.727	0.600	0.273	0.400	0.327	1.818	0.455	0.605	0.723
4.6	27585 (35.11)	0.648	0.712	0.352	0.288	0.360	2.250	0.494	0.549	0.789
4.7	20454 (26.03)	0.577	0.807	0.423	0.193	0.384	2.990	0.524	0.512	0.844
4.8	14554 (18.52)	0.505	0.882	0.495	0.118	0.387	4.280	0.561	0.493	0.887
4.9	10122 (12.88)	0.444	0.938	0.556	0.062	0.382	7.161	0.593	0.515	0.919
5.0	7056 (8.98)	0.394	0.975	0.606	0.025	0.369	15.760	0.622	0.609	0.942
5.1	4794 (6.10)	0.349	1	0.651	0	0.349	—	0.651	1.000	0.959

FPR, false positive ratio; FNR, false negative ratio; PLR, positive likelihood ratio; NLR, negative likelihood ratio; PPV, positive predictive value; NPV, negative predictive value.

### The association between FPG cutoff value and GDM diagnosis according to maternal age categories

1,837 (9.6%) women were diagnosed as GDM for maternal age ≤25 years, 6,085 (15.9%) women for maternal age among 26 to 30 years, 3,880 (24.8%) women for maternal age among 31 to 35 years, and 1,766 (36.0%) women for maternal age >35 years, *P* < 0.001. To evaluate the FPG value of OGTT to establish the diagnosis of GDM at 24–28 weeks’ gestation based on different maternal age, the ROC curves are shown in Table 3. The area under the ROC curve of different maternal age was 0.76, 0.74, 0.74, and 0.75, respectively for maternal age ≤ 25, 26–30, 31–35, and >35 years groups.

**Table 3:** The receiver operation characteristic curve of the participants among different maternal age.

Age (years)	GDM (n/%)	No-GDM (n)	*P* value	Area Under ROC curve	95CI	SE	*P* value
≤25	1837 (9.6)	17272		0.76	0.748–0.775	0.007	<0.001
26–30	6085 (15.9)	32140	<0.001	0.74	0.730–0.746	0.004	<0.001
31–35	3880 (24.8)	11744		0.74	0.730–0.750	0.005	<0.001
>35	1766 (36.0)	3135		0.75	0.735–0.765	0.008	<0.001

GDM, gestational diabetes mellitus; ROC, receiver operation characteristic; CI, confidence intervals; SE, standard error.

As presented in Table 4, with the cutoff FPG value of 4.5 mmol/L, 70.4% of pregnant women aged less than 25 years were diagnosed to have GDM with specificity of 0.658. As well, with the FPG cutoff value of 4.5 mmol/L, 70.6% of pregnant women aged among 26 to 30 years were diagnosed as GDM with specificity of 0.601, and 74.9% of pregnant women aged among 31–35 years were diagnosed as GDM with specificity of 0.542. Besides, 77.6% of pregnant women aged more than 35 years were diagnosed as GDM with the specificity of 0.497.

**Table 4:** Fasting glucose plasma cutoff values of gestational diabetes mellitus diagnosis according to age categories.

Cutoff point (mmol/L)	≤ 25 years			26–30 years			31–35 years			> 35 years		
	At or above the value, n (%)	Sensitivity	1-Specificity	At or above the value, n (%)	Sensitivity	1-Specificity	At or above the value, n (%)	Sensitivity	1-Specificity	At or above the value, n (%)	Sensitivity	1-Specificity
4.0	17077 (89.37)	0.958	0.887	35198 (92.08)	0.966	0.912	14838 (94.97)	0.974	0.942	4719 (96.29)	0.989	0.948
4.1	15714 (82.23)	0.931	0.811	32978 (86.27)	0.938	0.849	14136 (90.48)	0.952	0.889	4567 (93.19)	0.971	0.910
4.2	13850 (72.48)	0.885	0.708	29828 (78.03)	0.897	0.758	13105 (83.88)	0.922	0.811	4287 (87.47)	0.948	0.833
4.3	11712 (61.29)	0.827	0.590	25993 (68.00)	0.841	0.649	11750 (75.20)	0.880	0.710	3943 (80.45)	0.908	0.746
4.4	9423 (49.31)	0.772	0.463	21575 (56.44)	0.779	0.524	10082 (64.53)	0.818	0.588	3475 (70.90)	0.851	0.629
4.5	7194 (37.65)	0.704	0.342	17123 (44.80)	0.706	0.399	8284 (53.02)	0.749	0.458	2948 (60.15)	0.776	0.503
4.6	5224 (27.34)	0.624	0.236	13037 (34.11)	0.626	0.287	6570 (42.05)	0.673	0.337	2434 (49.66)	0.704	0.380
4.7	3641 (19.05)	0.565	0.151	9515 (24.89)	0.554	0.191	5046 (32.30)	0.594	0.233	1938 (38.54)	0.623	0.267
4.8	2397 (12.54)	0.499	0.086	6725 (17.59)	0.488	0.117	3667 (23.47)	0.507	0.145	1475 (30.10)	0.541	0.166
4.9	1579 (8.26)	0.459	0.043	4592 (12.01)	0.428	0.062	2600 (16.64)	0.437	0.077	1093 (22.30)	0.461	0.089
5.0	1074 (5.62)	0.419	0.018	3087 (8.08)	0.381	0.024	1856 (11.88)	0.384	0.031	832 (16.98)	0.402	0.039
5.1	629 (3.62)	0.377	0	2061 (5.39)	0.339	0	1300 (8.32)	0.335	0	631 (12.87)	0.357	0

### The association between FPG cutoff value and GDM diagnosis according to pre-pregnancy BMI categories

1,511 (10.5%) women were diagnosed as GDM for pre-pregnancy BMI <18.5 kg/m^2^, 8,657 (16.6%) women for pre-pregnancy BMI among 18.5 to 23.9 kg/m^2^, 2,743 (29.1%) women for pre-pregnancy BMI among 24.0 to 27.9 kg/m^2^, and 657 (35.7%) women for pre-pregnancy BMI ≥28 kg/m^2^. According to pre-pregnancy BMI categories, the ROC curves are presented in Table 5. The area under the ROC curve of pre-pregnancy BMI categories was 0.66, 0.74, 0.81, and 0.83, respectively for pre-pregnancy BMI <18.5 (underweight), 18.5–23.9 (normal), 24.0–27.9 (overweight), and ≥28 kg/m^2^ (obesity) groups.

**Table 5:** The receiver operation characteristic curve of the participants among different BMI.

BMI (kg/m^2^)	GDM (n)	No-GDM (n)	*P* value	Area Under ROC curve	95CI	SE	*P* value
<18.5	1511 (10.5)	12841		0.66	0.647–0.680	0.008	<0.001
18.5–23.9	8657 (16.6)	43595	<0.001	0.74	0.731–0.744	0.003	<0.001
24.0–27.9	2743 (29.1)	6674		0.81	0.794–0.815	0.005	<0.001
≥28.0	657 (35.7)	1181		0.83	0.811–0.854	0.011	<0.001

BMI, body mass index; GDM, gestational diabetes mellitus; ROC, receiver operation characteristic; CI, confidence intervals; SE, standard error.

As presented in Table 6, with the FPG cutoff value of 4.5mmom/L, 54.9% of pregnant women with pre-pregnancy BMI less than 18.5 kg/m^2^ (underweight) were diagnosed as GDM with the specificity of 0.692. Meanwhile, with the cutoff FPG value of 4.5 mmol/L, 70.8% of pregnant women with pre-pregnancy BMI among 18.5 to 23.9 kg/m^2^ (normal) were diagnosed to have GDM with specificity of 0.597. In addition, with the cutoff FPG value of 4.5 mmol/L, 84.7% or 89.3% pregnant women with pre-pregnancy BMI among 24–27.9 kg/m^2^ (overweight) or ≥28.0 kg/m^2^ (obesity) were diagnosed as GDM with specificity of 0.485 and 0.391 respectively.

**Table 6:** Fasting glucose plasma cutoff values of gestational diabetes mellitus diagnosis according to body mass index categories.

Cutoff point (mmol/L)	<18.5 kg/m^2^			18.5–23.9 kg/m^2^			24–27.9 kg/m^2^			≥28.0 kg/m^2^		
	At or above the value, n (%)	Sensitivity	1-Specificity	At or above the value, n (%)	Sensitivity	1-Specificity	At or above the value, n (%)	Sensitivity	1-Specificity	At or above the value, n (%)	Sensitivity	1-Specificity
4.0	12677 (88.33)	0.932	0.878	48318 (92.47)	0.970	0.916	9036 (95.95)	0.989	0.948	1801 (97.99)	0.991	0.974
4.1	11557 (80.53)	0.879	0.797	45349 (86.79)	0.942	0.853	8729 (92.69)	0.981	0.905	1760 (95.76)	0.986	0.942
4.2	10042 (69.97)	0.815	0.686	41123 (78.70)	0.904	0.764	8212 (87.20)	0.962	0.835	1693 (92.11)	0.977	0.890
4.3	8323 (58.03)	0.728	0.563	35920 (68.74)	0.850	0.655	7557 (80.25)	0.935	0.748	1593 (86.67)	0.963	0.813
4.4	6499 (45.28)	0.639	0.431	29912 (57.25)	0.786	0.530	6684 (70.98)	0.894	0.634	1460 (79.43)	0.927	0.721
4.5	4788 (33.36)	0.549	0.308	23695 (45.35)	0.708	0.403	5760 (61.17)	0.847	0.515	1306 (71.06)	0.893	0.609
4.6	3402 (23.70)	0.457	0.211	17950 (34.35)	0.625	0.288	4781 (50.77)	0.785	0.394	1132 (61.59)	0.845	0.489
4.7	2246 (15.65)	0.370	0.131	13049 (25.06)	0.549	0.191	3845 (40.83)	0.720	0.280	955 (51.96)	0.798	0.365
4.8	1404 (9.78)	0.300	0.074	9101 (17.42)	0.476	0.114	2974 (31.58)	0.637	0.184	785 (42.71)	0738	0.254
4.9	846 (5.89)	0.255	0.036	6166 (11.80)	0.412	0.060	2214 (23.51)	0.570	0.097	638 (34.71)	0.680	0.162
5.0	504 (3.51)	0.216	0.014	4141 (7.93)	0.363	0.023	1692 (17.97)	0.511	0.043	512 (27.86)	0.635	0.080
5.1	288 (2.01)	0.191	0	2759 (5.28)	0.319	0	1257 (13.35)	0.335	0	380 (20.67)	0.578	0

## Discussion

Although it had been reported that FPG was a poor predictor for GDM later in pregnancy based on the FPG level decreases at the end of the first trimester and with a low sensitivity or poor specificity^24,25^, in this study, ROC curve analysis presented that FPG ≥4.5 mmol/L was the optimal threshold for predicting GDM, with a sensitivity of 72.7% and a specificity of 60.0%.

Our study showed that the diagnostic rate of GDM using 5.1 mmol/L as the cutoff FPG value based on the IADPSG criteria was only 35.7%. However, another study indicated that the IADPSG criteria diagnosed GDM using the FPG value (55%) alone for the complete Hyperglycemia and Adverse Pregnancy Outcome (HAPO) cohort study. Interestingly, the diagnostic rate of GDM using the FPG value (5.1 mmol/L) was 47% in Singapore^26^. Compared with these results, the diagnostic rate is lower in this study. It is clear that the FPG cutoff value of 5.1 mmol/L was to establish the diagnosis of GDM based on the IADPSG criteria was not possible in China. If we use these criteria, there is a bigger possibility that the diagnosis of GDM in some pregnant women will be missed. Any local adaptations of these criteria will have to be dependent on the exact local data of the population under consideration.

Additionally, the Ministry of Health in China published the GDM diagnostic criteria in 2011, which stated that if FPG value was greater than or equal to 5.1 mmol/L, immediately, the pregnant women can be diagnosed as having GDM and if FPG value was greater than or equal to 4.4 mmol/L and less than 5.1 mmol/L, the 75-g OGTT must be performed in pregnant women^22^. Our research found that if FPG level was greater than or equal to 5.1 mmol/L, immediately, pregnant women can be diagnosed as having GDM with a specificity of 100%. Additionally, if FPG value was less than or equal to 4.5 mmol/L, pregnant women can be ruled out in the diagnosis of GDM with a sensitivity of 72.7%. In view of the above results, we conclude that with the FPG values between 4.5 and 5.1 mmol/L, an optimal threshold to rule out or identify GDM in China. This threshold range will reduce the performance of a number of OGTTs by about 50.37%. Interestingly, one study reported that an FPG value of 4.4 mmol/L should be used as the optimal cutoff value, which could reduce the performance of a number of OGTTs by about 50.3% with the probability that the diagnosis of GDM to about 12.2% of pregnant women may be missed^15^. Although the threshold of FPG value is different, the proportion of avoiding performance of OGTT is similar.

The study had some advantages. The major advantage was that its data were derived from a registered system with a large sample size, which registered Chinese pregnant women who came from the countryside and city to avoid selection bias. Moreover, it was the first research to evaluate the sensitivity and specificity of FPG value to establish the diagnosis of GDM in China with registered data, which had strong public health significance.

The study also had some limitations. Firstly, this study is a retrospective design that included unavoidable selection bias. Secondly, all data were from the MBRX, and pre-pregnancy BMI of Chinese differs from other population; hence, there was a lack of data from other regions. Thirdly, the effects of other factors on incidence of GDM are unavailable, such as physical activity or dietary habit.

As a conclusion, FPG test, with a high sensitivity and specificity, is used as an ideal GDM screening tool for low-resource countryside that simplifies the demanding algorithm for the establishment of the diagnosis of GDM. Moreover, for maternal age and pre-pregnancy BMI categories, FPG ≥4.5 mmol/L could be as an optimal predictor for GDM in all groups with a high sensitivity, which could improve the diagnostic rate and reduce the health burden of the Chinese societies and families.

## Data Availability

Data are available upon request. Please contact Xue-jun Li, professor, xmlixuejun@163.com.
